# Determinants of visit-to-visit systolic blood pressure variability among Ghanaians with hypertension and diabetes mellitus

**DOI:** 10.4314/gmj.v57i1.5

**Published:** 2023-01

**Authors:** Fred S Sarfo, Nana K Ayisi-Boateng, Samuel B Nguah, Osei Sarfo-Kantanka, Collins Kokuro, Hanson Ababio, Yaw Adu-Boakye, Bruce Ovbiagele

**Affiliations:** 1 Kwame Nkrumah University of Science and Technology, Kumasi, Ghana; 2 University of California, San Francisco, USA

**Keywords:** Hypertension, Variability, stroke, risk factors, Africa

## Abstract

**Objective:**

To identify the determinants of systolic blood pressure variability (SBPV) among Ghanaians.

**Design:**

We undertook a secondary analysis of data collected in a prospective study

**Setting:**

The study involved patients with hypertension and or diabetes receiving care in five hospitals in Ghana

**Main outcome measures:**

We assessed determinants of SBPV among 2,785 Ghanaian patients. We calculated the standard deviation (SD) of systolic BP recordings of 3 to 10 visits per patient over 18 months as a measure of SBPV. A multivariate linear regression analysis was fitted to identify factors independently associated with risk visit-to-visit SBP standard deviation.

**Results:**

The mean SD of individual patient visit-to-visit SBP overall was 14.8± 6.3 mm Hg. Those with hypertension and diabetes had the highest SD of 15.4 ±6.2 mm Hg followed by 15.2 ±6.5 mm Hg among those with hypertension only and then 12.0 ± 5.2 mm Hg among those with diabetes only, p<0.0001. Factors independently associated with SBPV with adjusted β coefficients (95% CI) included age: 0.06 (0.03 – 0.08) for each year rise in age, eGFR -0.03 (-0.05 - -0.02) for each ml/min rise, low monthly income of <210 Ghana cedis 1.45 (0.43-2.46), and secondary level of education -1.10 (-1.69, -0.50). Antihypertensive classes were associated with SBPV, the strongest associations being hydralazine 2.35 (0.03 – 4.68) and Methyldopa 3.08 (2.39 – 3.77).

**Conclusion:**

Several socio-demographic and clinical factors are associated with SBPV. Future studies should assess the contribution of SBPV to CVD outcomes among indigenous Africans and identify actionable targets.

**Funding:**

Funding for this study was provided by MSD, Novartis, Pfizer, Sanofi (each a Participant Company) and the Bill and Melinda Gates Foundation (collectively, the Funders) through the New Venture Fund (NVF). FSS and BO are also supported by funding from the National Heart, Lung, and Blood Institute (R01HL152188).

## Introduction

The global burden of hypertension is enormous, with a staggering 1.4 billion individuals affected.^1^ The greatest burden of hypertension is borne by adult populations in low-and-middle-income countries where the rate of awareness, detection and control are abysmally low.^1^,^2^ Hypertension is an emergent public health catastrophe accounting for millions of deaths annually from ischemic heart disease, stroke and chronic kidney disease.^3^,^4^

An inherent characteristic of blood pressure is its variability. This variability is recognisable beat-by-beat within 24 hours monitoring and over long-term observation (weekly, monthly, or even yearly.^5^ Physiologic mechanisms such as central and reflex autonomic modulation, elastic property of the arterial vasculature, and a complex mix of humoral, rheological and emotional factors account for much of the short-term BP variability.^6^–^8^

There is ample evidence suggesting that BP variability over 24 hours provides modest prognostic information concerning cardiovascular and all-cause mortality, independently of mean BP.^9^,^10^ Longer-term variability in BP is, however, believed to be contributed to by adherence to medication and seasonal changes.^11^–^13^ There are emerging data suggesting that long-term visit-to-visit systolic BP variability also contributes significantly to the occurrence of cardiovascular events, especially stroke, cardiovascular deaths and all-cause mortality, independently of average systolic BP.^11^, ^14^–^22^ Commonly reported measures of systolic blood pressure dispersion to include standard deviation (SD), coefficient of deviation (CV), and variability independent of the mean (VIM), of which SD is the most often reported in the literature.

There are limited data on visit-to-visit systolic blood pressure variability (SBPV) among indigenous Africans. This is partly due to a paucity of studies on the determinants of BP control from prospective cohort studies in sub-Saharan Africa (SSA).^23^ Given the emerging prognostic significance of SBPV and the poor outcomes of hypertension in LMICs in SSA, such information would be of value for clinicians involved in the care of millions of patients with hypertension across the sub-continent. Furthermore, although the prognostic relevance of long-term blood pressure variability has been severally reported, the factors which account for this variability have not been adequately described in the literature. Our objective, therefore, was to assess the determinants of visit-to-visit SBPV among Ghanaian patients with hypertension and/or diabetes mellitus at five hospitals over 18 months of follow-up.

## Methods

### Study Design

This is a secondary analysis of data collected in a prospective cohort of patients with hypertension and diabetes receiving care in Ghanaian hospitals. The Committee on Human Research Publications and Ethics (CHRPE) of the Kwame Nkrumah University of Science and Technology gave approval (CHRPE/LPI/006/22) for secondary analysis of the data of an earlier study whose protocol has been published elsewhere.^24^ Briefly, the study was conducted at five hospitals in Ghana with hypertension and diabetes specialty and general clinics. The five study sites included the Agogo Presbyterian Hospital (APH), Atua Government Hospital (AGH), Komfo Anokye Teaching Hospital (KATH), Kings Medical Center (KMC) and the Tamale Teaching Hospital (TTH). These study sites were situated in the northern, middle, and lower belts of Ghana to reflect geographic distribution and included primary, secondary, and tertiary levels of health care.

### Recruitment of Study Participants

Participants were eligible if they were 18 years or older with a known diagnosis of hypertension and/or type II diabetes presenting for routine care. Participants were excluded if they had hypertensive urgency or emergency or had glycemic complications at initial contact for enrollment. Consecutive participants meeting eligibility criteria were enrolled at each site. Informed consent was obtained from all consecutively enrolled participants.

### Evaluation of Study Participants

Trained Research Assistants interviewed study participants and collected demographic information such as age, gender, educational attainment, employment status, number of dependents on monthly income and health expenditures. Information on lifestyle behaviours such as alcohol use, cigarette smoking, level of physical activities, frequency and daily quantities of fruits and vegetable consumption and table-added salt were also recorded. The duration of hypertension or diabetes diagnosis was noted. Compliance with hypertension treatment was assessed using the 14-item version of the Hill-Bone compliance to the high blood pressure therapy scale.^25^ Stroke was self-reported if a participant had ever experienced sudden onset of weakness or sensory loss on one side of the body, sudden loss of vision, or sudden loss of speech. Heart failure was self-reported if a participant had ever experienced shortness of breath on exertion, lying down and swelling of both feet. BP measurements were performed following a standardised operating procedure implemented across study sites. Anthropometric assessments performed by study nurses include measurement of weight and height for body mass index (BMI) derivation and waist circumference.

### Laboratory measurements

An International Organization for Standardization (ISO)-certified laboratory was contracted to analyse serum creatinine to calculate the estimated glomerular filtration rate using the CKD-EPI equation.

### Prospective evaluations

Study participants visited study sites every two months to have clinic BP measured for 18 months.

### Study Outcome

We calculated systolic blood pressure recordings' standard deviation (SD) of 3 to 10 visits per patient over 18 months of follow-up. Blood pressure measurements of study participants were obtained using an automated blood pressure monitor across all study sites (Omron HEM-907XL). Two consecutive systolic BP readings from the same arm taken 2 minutes apart were recorded and averaged for the present analysis.

### Statistical analysis

Participants' socio-demographic characteristics, vascular risk factors, medications, medical history and adherence were first compared by tertiles of the standard deviation of the systolic blood pressure. Means were compared using Analysis of Variance (ANOVA), and proportions were compared using Chi-squared tests. We investigated a host of potential factors for associations with visit-by-visit systolic BP variability (SBPV) based on a literature search, our understanding of the epidemiology of BP control and empirical evidence from our data. A multivariate linear regression analysis was fitted to identify factors independently associated with the risk visit-to-visit systolic BP standard deviation as the dependent variable.

Independent variables evaluated within domains and included the following socio-demographic factors: age (continuous variable), gender, location of residence, educational attainment, employment status, and monthly income were all categorical variables; lifestyle/behavioural factors: cigarette smoking, current alcohol use, and table added salt (categorical), physical activity, fruit intake, vegetable intake, and antihypertensive therapy adherence (continuous); patho-biologic factors: co-morbid diabetes, diagnoses of stroke, cardiac failure, classes of antihypertensive or antiglycemic agents (categorical variables) with a duration of hypertension diagnosis, number of antihypertensive medications, estimated glomerular filtration rate, waist circumference being continuous variables; and finally health system factors: availability of all prescribed antihypertensives on NHIS. In all analyses, two-tailed p-values <0.05 were considered statistically significant. Secondary analysis considering those with hypertension history only vs those with diabetes with or without hypertension was also performed using linear regression modeling. Model diagnosis and fit were assessed using residual plots analysis. Statistical analysis was performed using SAS 9.4.

## Results

We included 2,785 patients with follow-up blood pressure recordings in our analysis. The mean (SD) age of the entire cohort was 57.8 (12.2) years, with more females (77.7%) than males. The mean (SD) of systolic blood pressure standard deviation was 14.8 (6.3). Those with hypertension and diabetes had the highest SD of 15.4 (6.2) mm Hg followed by 15.2 (6.5) mm Hg among those with hypertension only and then 12.0 (5.2) mm Hg among those with diabetes only, p<0.0001.

The mean age (SD) increased with increasing tertile of SBPV being 54.8 (12) years for the lower tertile, 58.6 (11.8) years for the middle tertile and 60.1 (12.3) years for the upper tertile, p<0.0001. Other demographic characteristics with significant differences across the three tiers were educational attainment, employment status, and monthly income, as shown in Table 1.

**Table 1:** Comparison of demographic and clinical characteristics by tertiles of systolic BP variability

Characteristic	Lower Tertile (n=928)	Middle Tertile (n=928)	Upper Tertile (n=928)	Total n (%)	P-value
**Age, *mean (SD)***	54.8 (12)	58.6 (11.8)	60.1 (12.3)	57.8 (12.2)	< 0.001
**Female sex, *n (%)***	699 (75.3)	725 (78.1)	739 (79.5)	2163 (77.7)	0.084
**Residence**					0.758
rural	326 (35.1)	331 (35.7)	316 (34)	973 (34.9)	
Semi-urban	215 (23.2)	203 (21.9)	200 (21.5)	618 (22.2)	
Urban	387 (41.7)	394 (42.5)	413 (44.5)	1194 (42.9)	
**Educational level**					< 0.001
No formal education	278 (30)	311 (33.5)	380 (40.9)	969 (34.8)	
Primary level	160 (17.2)	179 (19.3)	138 (14.9)	477 (17.1)	
Secondary level	377 (40.6)	340 (36.6)	315 (33.9)	1032 (37.1)	
Tertiary level	113 (12.2)	98 (10.6)	96 (10.3)	307 (11)	
**Employment**					< 0.001
Employed	687 (74)	626 (67.5)	573 (61.7)	1886 (67.7)	
Unemployed	241 (26)	302 (32.5)	356 (38.3)	899 (32.3)	
**Income**					< 0.001
>1,000 GHc	80 (8.6)	84 (9.1)	46 (5)	210 (7.5)	
210–1000 GHc	268 (28.9)	249 (26.8)	224 (24.1)	741 (26.6)	
<210 GHc	338 (36.4)	337 (36.3)	381 (41)	1056 (37.9)	
Don't know	242 (26.1)	258 (27.8)	278 (29.9)	778 (27.9)	
Medication cost covered by the NHIS, n (%)					0.341
All medications paid for	504 (54.3)	487 (52.5)	473 (50.9)	1464 (52.6)	
Not all medications paid for	424 (45.7)	441 (47.5)	456 (49.1)	1321 (47.4)	
**Disease class**					< 0.001
Hypertension only	472 (50.9)	513 (55.3)	543 (58.4)	1528 (54.9)	
Diabetes mellitus only	195 (21)	100 (10.8)	63 (6.8)	358 (12.9)	
Hypertension and Diabetes mellitus	261 (28.1)	315 (33.9)	323 (34.8)	899 (32.3)	
**Ever Smoked *n (%)***	55 (5.9)	66 (7.1)	64 (6.9)	185 (6.6)	0.552
**Alcohol intake, *n (%)***	68 (7.3)	83 (8.9)	63 (6.8)	214 (7.7)	0.191
**Salt added to food, *n (%)***	183 (19.7)	132 (14.2)	148 (15.9)	463 (16.6)	0.005
**Physical activity, *n (%)***	321 (34.6)	344 (37.1)	371 (39.9)	1036 (37.2)	0.058
**Hours spend exercising per week, *mean*** ***(SD)***	21.6 (25.7)	19.8 (23)	18.6 (23.2)	20 (24)	0.026
**Days of Fruit intake in past week, *mean*** ***(SD)***	2.7 (2.1)	2.5 (2)	2.5 (2)	2.6 (2)	0.037
**Fruit servings per day, *mean (SD)***	1.7 (1.7)	1.6 (1.4)	1.6 (1.4)	1.7 (1.5)	0.414
**Days of vegetables intake in past week,** ***mean (SD)***	5 (2.2)	5 (2.1)	4.9 (2.2)	5 (2.1)	0.314
**Vegetable servings per day, mean *(SD)***	2.2 (1.6)	2.2 (1.4)	2.2 (1.4)	2.2 (1.5)	1.00
**Heart failure, *n (%)***	59 (6.4)	48 (5.2)	50 (5.4)	157 (5.6)	0.498
**Stroke, n *(%)***	38 (4.1)	50 (5.4)	56 (6)	144 (5.2)	0.159
**Body Mass Index (Kg/m^2^), *mean (SD)***	26.4 (5.4)	26.8 (5.7)	26.6 (5.8)	26.6 (5.6)	0.374
**Waist Circumference, *mean (SD)***	95.2 (12.9)	96.3 (12.2)	95.7 (13.8)	95.7 (13)	0.191
**eGFR (ml/min/1.73 m^2^), *mean (SD)***	80.1 (13.1)	76.2 (16.2)	74 (17.4)	76.8 (15.9)	< 0.001
**Years since diagnosed hypertension,** ***mean (SD)***	7.1 (6.4)	8.1 (7)	8.8 (7.9)	8 (7.2)	< 0.001
**Years since diagnosed Diabetes, *mean*** ***(SD)***	8.1 (6.1)	9.6 (6.8)	10.6 (7.7)	9.4 (6.9)	< 0.001
**ACE-Inhibitors, *n (%)***	320 (34.5)	407 (43.9)	426 (45.9)	1153 (41.4)	< 0.001
**Angiotensin receptor blocker, *n (%)***	215 (23.2)	241 (26)	288 (31)	744 (26.7)	< 0.001
**Beta-Blockers, *n (%)***	52 (5.6)	76 (8.2)	112 (12.1)	240 (8.6)	< 0.001
**Calcium Channel Blockers, *n (%)***	577 (62.2)	628 (67.7)	668 (71.9)	1873 (67.3)	< 0.001
**Diuretic, *n (%)***	226 (24.4)	266 (28.7)	314 (33.8)	806 (28.9)	< 0.001
**Methyldopa, *n (%)***	75 (8.1)	120 (12.9)	218 (23.5)	413 (14.8)	< 0.001
**Hydralazine, *n (%)***	7 (0.8)	11 (1.2)	22 (2.4)	40 (1.4)	0.01
**Number of antidiabetic meds, *mean*** ***(SD)***	1.1 (1.2)	1 (1.2)	1 (1.2)	1 (1.2)	0.1
**Metformin, *n (%)***	420 (45.3)	387 (41.7)	372 (40)	1179 (42.3)	0.067
**Sulphonylurea, n (%)**	260 (28)	265 (28.6)	232 (25)	757 (27.2)	0.174
**Thiazolidinedione, n (%)**	167 (18)	175 (18.9)	169 (18.2)	511 (18.3)	0.881
**Insulin, n (%)**	148 (15.9)	109 (11.7)	111 (11.9)	368 (13.2)	0.011
**Dipeptidyl peptidase-4 inhibitor, n (%)**	3 (0.3)	1 (0.1)	1 (0.1)	5 (0.2)	0.448
**Statin, n (%)**	93 (10)	96 (10.3)	99 (10.7)	288 (10.3)	0.904
**Antiplatelet, n (%)**	87 (9.4)	98 (10.6)	104 (11.2)	289 (10.4)	0.427
**No. of Antihypertensives, *mean (SD)***	1.6 (1)	1.9 (1)	2.2 (1)	1.9 (1)	< 0.001
**Hillbone score, *mean (SD)***	18.1 (3.7)	18 (3.9)	18 (3.7)	18 (3.8)	0.841

ACE = Angiotensin Converting Enzyme; NHIS = National Health Insurance Scheme; eGFR = estimated glomerular filtration rate

### Factors associated with SBP variability in the entire cohort

In bivariate linear regression, we identified 24 variables with significant associations with SD of visit-by-visit systolic BP (Table 2). Upon adjustment for confounding variables, eleven remained significantly and independently associated with SBPV (shown in Table 2).

**Table 2:** Crude and adjusted associations between the demographic and clinical characteristics and blood pressure variability for all patients

Characteristic	Crude Coefficient (95%CI)	P-value	Adjusted Coefficient (95%CI)	P-value
Age in years	0.09 (0.07,0.11)	< 0.001	0.06 (0.03,0.08)	< 0.001
Male sex	-0.47 (-1.03,0.09)	0.101		
Residence				
rural	**Ref**		**Ref**	
Semi-urban	0.01 (-0.63,0.64)	0.981	-0.3 (-0.98,0.38)	0.385
Urban	0.56 (0.02,1.09)	0.04	0.24 (-0.36,0.83)	0.437
Educational level				
No formal education				
Primary level	-1.61 (-2.3,-0.92)	< 0.001	-1.12 (-1.82, -0.42)	0.002
Secondary level	-1.32 (-1.87,-0.77)	< 0.001	-1.10 (-1.69, -0.50)	< 0.001
Tertiary level	-1.43 (-2.23,-0.62)	< 0.001	-0.57 (-1.45,0.32)	0.210
Employment	1.27 (0.77,1.77)	< 0.001	-0.31 (-0.88,0.27)	0.300
Income				
>1,000 GHc				
210–1000 GHc	0.84 (-0.12,1.81)	0.086	1.05 (0.05,2.06)	0.039
<210 GHc	1.92 (0.99,2.86)	< 0.001	1.45 (0.43,2.46)	0.005
Don't know	1.85 (0.89,2.81)	< 0.001	1.62 (0.59,2.64)	0.002
Medication cost covered by the NHIS	0.27 (-0.2,0.74)	0.262		
Disease class*				
Hypertension				
Diabetes mellitus	-3.19 (-3.91,-2.48)	< 0.001		
Diabetes + hypertension	0.19 (-0.33,0.7)	0.477		
Ever Smoked	0.35 (-0.6,1.29)	0.47		
Alcohol intake	-0.2 (-1.08,0.68)	0.653		
Salt added to food*	-0.86 (-1.49,-0.24)	0.007		
Physical activity	0.43 (-0.05,0.92)	0.082		
Hours of exercise per week	-0.01 (-0.02,0)	0.012	0.00 (-0.01,0.01)	0.985
Days of Fruit intake per week	-0.1 (-0.21,0.02)	0.09		
Fruit servings per day	-0.02 (-0.17,0.14)	0.803		
Days of vegetables per week	-0.04 (-0.15,0.07)	0.48		
Vegetable servings per day	0.04 (-0.12,0.2)	0.59		
Heart failure	-0.53 (-1.55,0.49)	0.308		
Stroke	1.38 (0.32,2.44)	0.011	0.54 (-0.54,1.62)	0.326
Body mass index, per kg/m2 rise	0.01 (-0.03,0.06)	0.534		
Waist Circumference	0.01 (-0.01,0.03)	0.436		
eGFR, per ml/min rise	-0.06 (-0.08,-0.05)	< 0.001	-0.03(-0.05,-0.02)	< 0.001
Duration of hypertension*	0.08 (0.05,0.12)	< 0.001		
Duration of Diabetes*	0.12 (0.08,0.17)	< 0.001		
**Classes of antihypertensive medications**				
ACE-Inhibitors	1.04 (0.56,1.51)	< 0.001	1.49 (0.94,2.03)	< 0.001
Angiotensin receptor blocker	1.26 (0.74,1.79)	< 0.001	1.04 (0.43,1.65)	< 0.001
Beta-Blockers	2.19 (1.36,3.02)	< 0.001	1.97 (1.12,2.83)	< 0.001
Calcium Channel Blockers	1.19 (0.69,1.69)	< 0.001	0.68 (0.11,1.24)	0.019
Diuretic	1.4 (0.88,1.91)	< 0.001	1.2 (0.63,1.76)	< 0.001
Methyldopa	3.79 (3.14,4.43)	< 0.001	3.08 (2.39,3.77)	< 0.001
Hydralazine	4.3 (2.33,6.26)	< 0.001	2.35 (0.03,4.68)	0.048
Number of antidiabetics*	-0.24 (-0.44, -0.05)	0.014		
Metformin	-0.49 (-0.96,-0.01)	0.045	0.57 (-0.3,1.44)	0.199
Sulphonylurea	-0.48 (-1.01,0.05)	0.074	-0.58 (-1.47,0.31)	0.201
Thiazolidinedione	-0.19 (-0.79,0.42)	0.544	0.2 (-0.53,0.94)	0.586
Insulin	-0.96 (-1.65,-0.27)	0.007	0.08 (-0.89,1.05)	0.873
DPP4-inhibitor	-3.15 (-8.69,2.4)	0.266		
Statin	0.31 (-0.46,1.08)	0.437		
Antiplatelet	0.93 (0.16,1.7)	0.018	0.61 (-0.21,1.43)	0.143
No. of Antihypertensive*	1.66 (1.44,1.88)	< 0.001		
Hillbone score	-0.01 (-0.08,0.06)	0.809		

ACE = Angiotensin Converting Enzyme; NHIS = National Health Insurance Scheme; eGFR = estimated glomerular filtration rate; DPP4 = Dipeptidyl peptidase-4

### Sensitivity analyses:

**(a) Factors associated with SBP variability among hypertensives only:** Seven (7) out of 16 factors identified in bivariate analyses remained independently associated with SBPV. These include increasing age, educational attainment, eGFR, ACE-I use, beta-blocker, methyldopa, and antiplatelet use (Table 3).
**(b) Factors associated with SBP variability among diabetics:** Among diabetics (including those with or without hypertension diagnosis) five out of 20 factors identified in bivariate analyses remained significantly associated with SBPV after adjustment in a multivariate linear regression model. These include increasing age, monthly income, duration of hypertension diagnosis, ARB use, and methyldopa use (not shown).

**Table 3:** Crude and adjusted associations between the demographic and clinical characteristics and blood pressure variability for patients with hypertension

Characteristic	Crude coefficient (95% CI)	P-value	Adjusted coefficient (95% CI)	P-value
Age in years	0.06 (0.03,0.08)	< 0.001	0.04 (0.01,0.08)	0.015
Female sex	-0.15 (-0.94,0.64)	0.714		
Residence				
Rural				
Semi-urban	0.72 (-0.14,1.59)	0.102	-0.12 (-1.07,0.82)	0.797
Urban	1.11 (0.37,1.85)	0.003	0.4 (-0.43,1.22)	0.345
Educational level				
No formal education				
Primary level	-1.38 (-2.33,-0.42)	0.005	-1.36 (-2.35,-0.37)	0.007
Secondary level	-1.1 (-1.86,-0.34)	0.005	-1.47 (-2.3,-0.64)	< 0.001
Tertiary level	-1.96 (-3.08,-0.84)	< 0.001	-1.7 (-2.96,-0.43)	0.009
Employment	1.07 (0.36,1.78)	0.003	-0.2 (-1.04,0.64)	0.635
Income				
>1,000 GHc				
210–1000 GHc	0.59 (-0.86,2.04)	0.425	0.49 (-1.05,2.03)	0.535
<210 GHc	1.52 (0.13,2.9)	0.032	0.73 (-0.81,2.27)	0.353
Don't know	1.79 (0.34,3.23)	0.015	0.79 (-0.79,2.37)	0.325
Medication cost covered by the NHIS	0.28 (-0.38,0.93)	0.409		
Ever Smoked	0.91 (-0.51,2.33)	0.208		
Alcohol intake	-0.11 (-1.26,1.03)	0.845		
Salt added to food	-0.79 (-1.64,0.07)	0.071		
Physical activity	0.4 (-0.26,1.07)	0.236		
Hours of exercise per week	-0.01 (-0.03,0)	0.11		
Days of Fruit intake per week	-0.08 (-0.24,0.09)	0.352		
Fruit servings per day	0.01 (-0.19,0.2)	0.936		
Days of vegetables per week	0.02 (-0.13,0.17)	0.802		
Vegetable servings per day	0.19 (-0.03,0.41)	0.089		
Heart failure	-0.58 (-1.88,0.73)	0.385		
Stroke	1.8 (0.31,3.3)	0.018	1.07 (-0.53,2.66)	0.19
Body mass index	-0.05 (-0.1,0.01)	0.086		
Waist Circumference	-0.02 (-0.04,0.01)	0.137		
eGFR	-0.07 (-0.09,-0.04)	< 0.001	-0.04 (-0.07,-0.02)	< 0.001
Duration of hypertension	0.05 (0,0.09)	0.042	-0.03 (-0.08,0.02)	0.29
ACE-Inhibitors	1.35 (0.69,2.01)	< 0.001	1.47 (0.28,2.65)	0.016
Angiotensin receptor blocker	0.91 (0.15,1.67)	0.018	0.39 (-0.82,1.61)	0.525
Beta-Blockers	2.02 (1.02,3.02)	< 0.001	1.50 (0.16,2.85)	0.029
Calcium Channel Blockers	-0.59 (-1.5,0.32)	0.205		
Diuretics	0.85 (0.19,1.5)	0.011	0.68 (-0.42,1.78)	0.226
Methyldopa	3.17 (2.32,4.03)	< 0.001	2.11 (0.9,3.32)	< 0.001
Hydralazine	5.1 (2.25,7.95)	< 0.001	3.11 (-1.51,7.72)	0.187
Statin	0.66 (-0.87,2.18)	0.398		
Antiplatelet	1.69 (0.41,2.97)	0.009	1.42 (0.08,2.76)	0.038
No. of Antihypertensives	1.68 (1.33,2.04)	< 0.001	0.4 (-0.57,1.38)	0.417
Hillbone score	0.03 (-0.05,0.12)	0.429		

ACE = Angiotensin Converting Enzyme; NHIS = National Health Insurance Scheme; eGFR = estimated glomerular filtration rate;

## Discussion

Long-term blood pressure variability assessed using visit-to-visit BP measures has emerged to be strongly and independently associated with subclinical organ damage, cardiovascular events, all-cause mortality, and chronic kidney disease progression.^11^,^14^–^22^ In this prospective study among individuals living with hypertension and/or diabetes receiving care across five hospitals in Ghana, we found that the average standard deviation of individual patient visit-to-visit systolic blood pressure overall was 14.8 (6.3) mm Hg. SBPV was highest among hypertensives with co-morbid diabetes, followed by those with hypertension and diabetes only. Our measures of blood pressure variability are largely compatible with data from previous studies among hypertensives or individuals at high CVD risk.

For instance, the SD among a cohort of Japanese with hypertension was 13.7 mm Hg 26, hypertensives from Sri Lanka had an SD of 13.1 mmHg^27^, and one European cohort from Scotland, Ireland, and the Netherlands reported an SD of 14.1 mmHg among elderly patients at high risk for CVD.^21^ In contrast, the standard deviation of SBP obtained among a general population in two US studies was lower at 7.7 mm Hg and 9.0 mm Hg, respectively.^11^,^18^ Three Italian studies among patients with end-stage kidney disease reported standard deviations of 9.7 mmHg, 10.9 mmHg and 11.0, respectively.^16^,^17^,^19^ The high variability of systolic BP among hypertensives on treatment, beyond mean blood pressures, may be a potential reason for the potent associations between hypertension and CVD events such as stroke, as suggested by Rothwell and colleagues.^28^,^29^

Meta-analytic data suggest that each 1-mmHg rise in systolic BP standard deviation is associated with a 3% [95% CI: 2-4%] rise in hazard of all-cause mortality, a 10% [95% CI: 2-17%] rise in hazard of cardiovascular mortality and 2% [95% CI: 1-3%] rise in hazard of stroke.^22^ Given the significant impact of systolic BP variability on events and outcomes independently of age and mean systolic BP, elucidating the factors associated with this emerging vascular risk factor seems warranted. As a measure of the dispersion of blood pressure, visit-to-visit variations in blood pressure are thought to reflect seasonal changes, measurement errors and adherence to antihypertensive medications.^22^ However, in our study, adherence was not associated with SBPV. One potential explanation for this is that baseline adherence may have changed during follow-up; hence one-time assessment of adherence to therapy may not serve as a good proxy for subsequent adherence during follow-up. An additional reason could be that the Hill-Bone questionnaire, which assesses three domains, medication adherence, adherence to salt intake and clinic appointment, may generate a composite score that does not directly contribute to long-term visit-by-visit variability. Finally, there could be residual confounding due to interactions between adherence and other covariates, which we could not extricate in our analyses.

We identified 11 socio-demographic, clinical and medication-related factors independently associated with SBPV in this sample of Ghanaians with hypertension and/or diabetes. Among the socio-demographic covariates, age demonstrated a strong and consistent association with the SD of SBP. This association between increasing age and high SBP variability is thought to be mediated by arterial stiffness exacerbated by ageing.^30^ We also found evidence to suggest that lower socio-economic indices such as lower educational attainment and lower income were incrementally and independently associated with much higher variability on SBP. This is noteworthy given the well-established links between the increased risk of hypertension among those with the lowest socio-economic status indicators, such as income, occupation, and education in high-income countries.^31^ The situation in sub-Saharan Africa with pervasive and endemic low socio-economic status is even dire, with 44% to 93% of hypertensive patients being unaware of their disease, only 18% on medications and abysmally, with 7% achieving adequate blood pressure control.^32^ We extend these findings in the present study by showing that lower socio-economic status may predispose individuals to wider fluctuations in blood pressure, a harbinger of adverse cardiovascular events in these vulnerable populations.

A dose-dependent correlation between the estimated glomerular filtration rate and systolic BPV was noted in the present study. The eGFR declined with the rising tertile of SBPV such that eGFR at the lower tertile was 80.1 ml/min, the middle tertile was 76.2 ml/min, and the higher tertile was 74ml/min, p<0.0001. A previous study among nearly 16,500 Spaniards with hypertension with and without chronic kidney disease showed an increase in short-term SBP variability across all the stages of kidney disease.^33^ The associations between baseline eGFR and long-term SBP variability may be induced by arterial stiffening in chronic kidney disease (CKD). Chronic kidney disease is quite prevalent among Ghanaians.^34^,^35^ Indeed, among Ghanaians with diabetes and hypertension, there is a gradient in the prevalence of CKD from 28.5% among those with hypertension and diabetes to 26.3% among those with hypertension only and 16.1% among those with diabetes only.^34^ With the progressive decline in renal function, two distinct pathologic types of vascular calcifications ensue, i.e. intimal calcifications, manifest as atherosclerotic plaques and medial calcification due to abnormal deposition of calcium phosphate in the arterial media.^36^,^37^ Unlike intimal calcifications, medial calcification, accelerated by renal impairment, does not obstruct the vascular lumen but promotes arterial stiffening resulting in increased pulse pressure and sheer stress.^38^

As expected, all antihypertensive drug classes were significantly associated with SBPV, with calcium channel blockers exerting the least variability, while alpha methyldopa had the most profound effect on SBPV in the entire cohort. Among those with hypertension alone on therapy, using calcium channel blockers (CCB,) non-loop diuretics, and angiotensin receptor blockers (ARBs) had non-significant associations with SBPV. A meta-analysis of 398 trials performed by Webb and colleagues demonstrated that CCBs and thiazide-like diuretics attenuated interindividual BPV, whilst ACE inhibitors, ARBs, and beta blockers tended to increase BPV over the long term.^39^ Though the mechanisms for the salutary effects of long-acting non-dihydropyridine CCBs on BPV have not been fully elucidated, their vasodilatory effects on the arterial beds and possibly via improved arterial compliance, baroreceptor function and autonomic nervous system modulation may partly explain these observations. In our study, we tested the effect of each class of antihypertensive for its association with SBPV, although almost all patients were on two or more combinations of antihypertensive medications. A high prevalence of resistant and refractory forms of hypertension is observed among Ghanaians, especially among stroke survivors requiring further investigation.^40^–^43^

There are limitations to this study worth noting. Blood pressure recordings were taken every two months at five medical centres for 18 months of follow-up with a potential for measurement errors. To mitigate this, we procured a validated automated BP monitor (OMRON^®^) to be deployed across all sites, and study nurses were trained on a protocol for BP measurements with quality checks. This study lacks associations between SBPV and outcomes such as incident stroke or mortality due to the relatively short follow-up and lack of data on mortality. Although stroke was associated with SBPV on bivariate analysis, this association attenuated into non-significance upon adjustment for covariates. We have, however, previously reported on determinants of incident stroke in this cohort.^44^–^46^ This study to the best of our knowledge is one of the few studies from sub-Saharan Africa to investigate the determinants of SBPV among a cohort of hypertensives and diabetics receiving healthcare at primary, secondary and tertiary levels of care.

## Conclusion

An array of socio-demographic factors, namely increasing age, educational attainment, monthly income and clinical factors, including renal function and antihypertensive medications, exerted a differential effect on inter-individual systolic blood pressure variability among a sample of Ghanaians with hypertension and or diabetes mellitus. Further studies are required to assess the impact on CVD outcomes of this emerging hypertension phenotype among indigenous Africans.
